# Silkworm pupae as source of high‐value edible proteins and of bioactive peptides

**DOI:** 10.1002/fsn3.1546

**Published:** 2020-05-16

**Authors:** Alessandra Anna Altomare, Giovanna Baron, Giancarlo Aldini, Marina Carini, Alfonsina D'Amato

**Affiliations:** ^1^ Department of Pharmaceutical Sciences Università degli Studi di Milano Milan Italy

**Keywords:** bioactive peptides, *Bombyx mori*, high‐resolution mass spectrometry, protein profiling, semiquantitative analyses

## Abstract

To characterize the high‐value protein content and to discover new bioactive peptides, present in edible organisms, as silkworm pupae, semiquantitative analytical approach has been applied. The combination of appropriate protein extraction methods, semiquantitative high‐resolution mass spectrometry analyses of peptides, in silico bioactivity and gene ontology analyses, allowed protein profiling of silkworm pupae (778 gene products) and the characterization of bioactive peptides. The semiquantitative analysis, based on the measurement of the emPAI, revealed the presence of high‐abundance class of proteins, such as larval storage protein (LSP) class. This class of proteins, beside its nutrient reservoir activity, is of great pharmaceutical interest for their efficacy in cardiovascular diseases. Potential allergens were also characterized and quantified, such as arginine kinase, thiol peroxiredoxin, and Bom m 9. This powerful bioanalytical approach proved the potential industrial applications of *Bombyx mori* pupae, as source of high‐value proteins in a green and “circular” economy perspective.

## Graphical abstract

Protein content by the semiquantitative analyses of *Bombyx mori* pupae. Identification of high‐value bioactive peptides by mass spectrometry. Potential industrial applications of *B. mori* pupae, as nutrient reservoir.

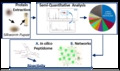

## 1 INTRODUCTION

Insects are a source of essential nutrients, such as proteins, fat, minerals, and vitamins, and of micronutrients, such as copper, iron, manganese, and riboflavin. Silkworms (*Bombyx mori*), including mulberry and non‐mulberry silkworm pupae, are a typical Asian food consumed from ancient times due to their high protein content. Silkworm pupae, which accounts for approximately 60% of the cocoon weight, are often discarded as waste material or used as fertilizer after the reeling process, even though they contain high percentages of proteins (18 kinds of amino acids have been identified, eight of them essential for man; Yang, Tang, Tong, & Liu, 2009) and fats (mainly unsaturated fatty acids; Rao, 1994).

An increased food/feed requirement in the next years can be easily predicted by considering the increasing population in the world, estimated around 9 billion people toward 2050. Taking into account the even more relevant reduction in biodiversity resources, edible insects, a traditional food all over the world owing to their high protein, can be considered a potential source for production of protein, either for direct human consumption, or indirectly in recomposed foods (with extracted protein from insects), as a protein source into feedstock mixtures (Rumpold & Schlüter, 2013) or as a source of bioactive peptides for applications in industry (Lemes et al., 2016). Silkworm pupae peptides have several potential functionality activities, as antiadipogenesis in rat adipocytes (Lee et al., 2012), and antimicrobial activity (Cheng et al., 2006).

Following the reintroduction of sericulture in Italy, owing to the consolidated expertise of the CREA‐API excellence research center in Padua, the production of Italian silk is going to start again on an industrial scale, increasing production of pupae too. Due to this fact, the silk industry might induce a real “circular economy,” producing raw material for other high added value production processes in different industrial fields, as silk industry biorefineries.

*Bombyx mori* larvae contain 54% of proteins, 8% of fat, 6% of fiber, 6% of ash and energy content of 390 kCal/100 g, even if the quality of the insect protein content has to be assessed (Blasquez, Moreno, & Martinez Camacho, 2012). The feeding of rats with silkworm pupae resulted in a lower chemical score in comparison with high‐value proteins of egg or casein, probably due to the presence of an ecdysone that confers a bad odor to the insects. Other studies showed a high content of essential amino acids, such as 77% of Lys and 70% of Leu in pupae (Rumpold & Schlüter, 2013). Another aspect to consider is the presence of potentially protein allergens, such as Bom m 9, chitinase, and paramyosin, and thiol peroxiredoxin that induce allergic asthma (Wang et al., 2016; Zhao, Li, Kuang, Luo, & Li, 2015; Zuo, Lei, Yang, & Liu, 2015).

Bioactive peptides are peptides showing functional properties, as antimicrobial, antihypertensive, antioxidative, and immune‐modulatory activities. They are produced from food matrices, as edible organisms, during gastrointestinal digestion and they can interact with human body as nervous, cardiovascular or gastrointestinal system. In addition, bioactive peptides can be used in food or cosmetic industry for antioxidative and antimicrobial activity (Lemes et al., 2016).

More research studies are requested to assess the protein profiling, the discovery of bioactive peptides and eventually allergens in silkworm pupae.

The aim of this work was to gain a deeper insight into the silkworm nutritional/functional quality of the components which can be extracted from insects, by the combined semiquantitative mass spectrometry‐based approach.

## 2 MATERIALS AND METHODS

### 2.1 Silkworm pupae treatment

The *B. mori* pupae were obtained by breeding on mulberry leaf polyhybrid larvae, produced starting from four productive strains preserved at the CREA‐API silkworm germplasm bank. First crosses were obtained by crossing two Japanese and two Chinese strains. First crosses were then crossed between each other to yield the polyhybrid eggs. After incubation of the eggs, the newly hatched larvae were bred according to best practice in silkworm rearing (temperature, relative humidity, and photoperiod). Afterward, pupae were air‐dried at 60°C until constant weight. Proteins were precipitated from a sample of silkworm pupae lyophilized at our disposal (provided by CREA‐API company) using two alternative protocols: aqueous extraction (Yi et al., 2013) and Folch extraction (Milkovska‐Stamenova & Hoffmann, 2016). Fifty grams of powder were dissolved in 150 ml of water and then the mixture was shuffled for 3 min by using a blender (Braun multiquick 3), to create a dispersion as much homogeneous as possible. This dispersion was sifted through a sieve with a porosity of 500 μm in order to remove any incompletely fragmented particles; 5 ml of the filtered sample was collected for the protein extraction, while the remaining 20 ml were further sifted on 125 µm pore size filter; and 5 ml were stored for protein extraction and the remaining 10 ml filtered on 40 µm pore size filter.

### 2.2 Aqueous extraction

In aqueous fractionation, the dispersion was centrifuged (centrifuge Rotina 380 R; Hettich) at 15,000 *g* for 30 min at 4°C, after filtration. The three fractions, precipitate, supernatant, and fatty phase, were collected. To improve the purity, the fatty phase was aspirated with a syringe while the remaining fractions were further centrifuged. The final supernatant and the precipitate (pellet) were easily separated.

The pellet was dried under nitrogen flow and weighted, dissolved in the extraction buffer, formed by 50 mM Tris‐HCl pH 8.0, 200 mM DTT, 0.3% SDS (Bio‐Rad Laboratories) to obtain a concentration of 100 mg/ml, and incubated on a rotator overnight at room temperature. After the incubation the mixture was centrifuged at 14,000 *g* for 1 hr at 4°C, and the resulting supernatant and the aqueous supernatant were subjected to trichloroacetic acid (TCA) precipitation (10% w/v; Sigma‐Aldrich). The solutions were mixed, incubated on ice for 30 min and centrifuged (10 min, 14,000 *g*, 4°C). The obtained precipitates were washed three times with ice‐cold acetone (1:1 v/v), and finally suspended in solubilization buffer (50 mM Tris‐HCl pH 7.2, 50 mM NaCl) to obtain a 100 mg/ml solution. Proteins were quantified by a colorimetric Bradford assay using BSA as standard.

### 2.3 Folch extraction

Proteins were purified in duplicate from the sifted raw materials (at different pore size sieves: 500/125/40 µm) using Folch extraction (methanol/chloroform/water). Methanol (7.5 ml) and chloroform (15 ml; Sigma‐Aldrich) were added to 1 ml of each sample and the mixture was continuously shaken for 1 hr at 4°C, and then cold water (12.5 ml) was added. After 10 min, the samples were centrifuged (10 min, 10,000 *g*, 4°C), the organic phase collected, and evaporated under vacuum. The remaining aqueous phase was centrifuged again (10 min, 10,000 *g*, 4°C), collected, and immediately dried under vacuum, while the protein pellets were dried under nitrogen flow. The dried protein pellets were weighted, dissolved in extraction buffer (50 mM Tris‐HCl pH 8.0, 200 mM DTT, 0.3% SDS) to a final concentration of 100 mg/ml and incubated on a rotator overnight at room temperature. Then, all protein samples were centrifuged at 11,000 rpm for 1 hr at 4°C, and the resulting supernatant collected for TCA precipitation, as described above. Proteins were quantified by a colorimetric Bradford assay using BSA as standard.

### 2.4 One‐dimensional analysis (SDS‐PAGE)

Protein separation was performed under reducing conditions; aliquots of 25 µg of proteins were mixed with Laemmli sample buffer (containing 50 mM DTT) and denatured at 95°C for 5 min. Samples and the standard proteins mixture (Precision Plus Protein Standards, Bio‐Rad Laboratories) were separated on precast gels (Any KD Mini Protean TGX; Bio‐Rad Laboratories), at 200 V. Gels were stained using Coomassie blue (Bio‐Safe G250; Bio‐Rad Laboratories). Images were acquired using the GS‐800 densitometer and analyzed by Quantity One software (Bio‐Rad Laboratories).

### 2.5 Protein in‐gel digestion

Protein bands were excised from gels, finely chopped, and washed with 200 µl of 50 mM ammonium bicarbonate (Bio‐Rad Laboratories). Gel pieces were destained using solution of 25 mM NH_4_HCO_3_/ACN, 1/1, v/v (Sigma‐Aldrich) following by acetonitrile (ACN). Proteins were reduced by 10 mM DTT at 56°C for 1 hr and then alkylated by 55 mM iodoacetamide at room temperature for 45 min in the dark. In‐gel digestion was performed by overnight‐incubation at 37°C with 1 µg of sequencing‐grade trypsin (Roche) in 50 mM ammonium bicarbonate. Then, the supernatants were collected and acidified with formic acid up to a final concentration of 1%. And the peptides were extracted following the method of previous published paper (Colzani et al., 2016).

### 2.6 Mass spectrometry analyses

The extracted peptides were analyzed by nLC‐MSMS, using an nHPLC, UltiMate 3000 RSLCnano System, connected to an LTQ‐Orbitrap XL mass spectrometer (Thermo Scientific Inc.). The peptide mixtures were separated by reversed‐phase chromatography (C_18_HALO PicoFrit column, 75 μm x 10 cm, 2.7 μm particles, pores 100 Å; New Objective*)* by 55 min linear gradient (1%–35% of ACN, 0.1% formic acid, water). The data were acquired in data‐dependent mode (DDA) by Xcalibur software (version 2.0.7; Thermo Scientific Inc.). All the settings were selected as in previous publications (Altomare et al., 2016; Marchis et al., 2017).

### 2.7 Data processing using different bioinformatics tools

#### 2.7.1 Protein identification

The identification of proteins was obtained consulting Uniprot_*Bombix Mori* database (22,971 entries) by Sequest algorithm in Proteome Discoverer software (version 1.3; Thermo Scientific). The mass tolerance was set as 5 ppm and fragment tolerance as 0.5 Da in the searching. Cysteine carbamidomethylation (+57.021 Da) was set as fixed modification, methionine oxidation (+15.995 Da) as variable modification and trypsin as proteolytic enzyme. The Decoy database was consulted to calculate the false discovery rate. The identifications were accepted only with FDR of 0.01 and at least two identified peptides (Altomare et al., 2016).

#### 2.7.2 Semiquantitative analysis

To estimate the protein contents in the complex mixtures, a semiquantitative analysis, based on emPAI was applied (Ishihama et al., 2005). Briefly protein abundance index (PAI) represents the number of observed peptides divided by the number of observable peptides per protein. To calculate the number of observable peptides per protein, proteins were digested in silico, exploiting the PeptideMass tool in Uniprot website (http://web.expasy.org/cgi‐bin/peptide_mass/peptide‐mass.pl?P02768), adjusted to simulate the experimental conditions. The number of observed peptides per protein, a method of counting unique parent ions was used (Ishihama et al., 2005).

The PAI is defined as (Equation 1):

(1) $$\text{PAI}=\frac{N_{\text{obsd}}}{N_{\text{obsl}}}$$

where *N*_obsd_ and *N*_obsbl_ are the number of observed peptides per protein and the number of observable peptides per protein, respectively. For absolute quantitation, PAI was converted to exponentially modified PAI (emPAI), defined as follows (Equation 2):

(2) $$\text{emPAI}=10^{\text{PAI}}-1$$

which is proportional to protein content in a protein mixture.

Thus, the protein contents in molar fraction percentages are described as (Equation 3):

(3) $$\text{Protein content}\;\left(\text{mol}\;\;\%\right)=\frac{\text{emPAI}}{\sum \left(\text{emPAI}\right)}\times 100$$

All the percentage values related to the protein contents were additionally normalized on the basis of the PSM (peptide spectral match) count, in order to consider the abundance of each peptide, strictly related to the number of spectra matched by the software during the data elaboration.

Equations 2 and 3 were modified as below reported:

$$\text{emPAI}\;\text{normalized}\;\left(\text{PSM}\right)=\frac{10\text{PAI}-1}{\sum \left(\text{PSM}\right)}\times \text{PSM}$$

$$\text{Protein}\;\text{Content}\;\left(\text{mol}\%\right)\;\text{normalized}\;\left(\text{PSM}\right)=\frac{\text{emPAInorm}}{\sum \left(\text{emPAInorm}\right)}\times 100.$$

#### 2.7.3 Generation of protein–protein interaction network

The protein–protein interaction network was built using all identified proteins and selecting *B. mori* genome as a reference database by STRING software (v.9.1, http://stringdb.org/; Szklarczyk et al., 2017). GO, KEGG, PFAM, and InterPro terms were selected as functional annotations for the enrichment analyses. The K‐means algorithm in STRING was used to obtain cluster networks based on GO terms: cellular localization, molecular functions, biological processes; PFAM domain terms, and KEGG terms of found proteins. In addition, Gene Ontology enrichment analyses were performed by ClueGO, a plug in Cytoscape (Bindea et al., 2009).

#### 2.7.4 Ranking of bioactive peptides

The *B. mori* proteome was “in silico” digested by stomach (pepsin) and intestinal (trypsin, chymotrypsin, elastase, carboxypeptidase A and B, and aminopeptidases) enzymes. The analyses were performed by MS‐Digest application in the ProteinProspector software (v 5.10.1, http://prospector.ucsf.edu/prospector/mshome.htm. The resulted peptide sequences were further analyzed by PeptideRanker (http://bioware.ucd.ie/~testing/biowareweb/) to assign a biofunctionality score. The highest scored sequences were then compared with the collection of antimicrobial peptides (CAMP) database search the antimicrobial bioactive peptides (http://www.bicnirrh.res.in/antimicrobial/).

## 3 RESULTS

### 3.1 Protein extraction and SDS‐PAGE analysis of silkworm pupae

**FIGURE 1:**
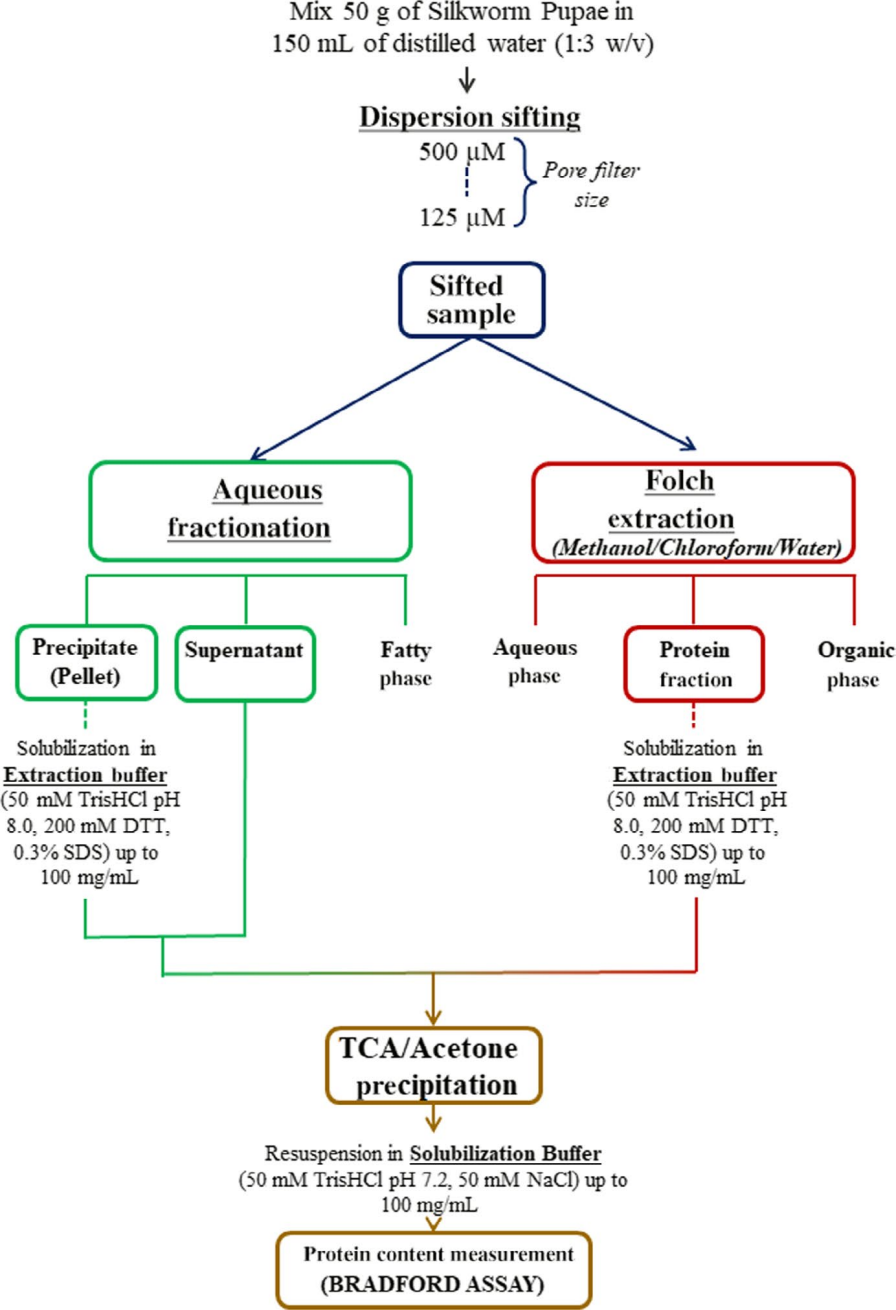
Flowchart of protein extraction protocol, showing the different methods used to purify proteins

Figure 1 shows the complete workflow setup for high‐yield protein extraction. In order to go more deeply in the knowledge of the *Bombix Mori* proteome, an experimental protocol, aimed to efficiently extract the protein fraction from dried silkworm pupae, was performed. After testing various extraction procedures reported in literature, the steps reported in the methods section, were applied allowing the most heterogeneous protein extraction. The final combination of TCA and cold acetone precipitation was used to precipitate proteins and to remove any remained contaminants. In Table 1, is reported the total amount of proteins extracted by using the combination of the different methods previously described, quantified by the Bio‐Rad protein assay.

**TABLE 1:** Results of Bradford Assay of protein lysates, obtained by different protocols

	Weight of raw material (g)	Resuspension volume after TCA precipitation (ml)	Protein concentration—(mg/ml)	Protein extraction (mg)
Pellet (Aqu. Extr.)	3.67 (N_2_ dried)	2	13.11	26.21
Supernatant (Aqu. Extr.)	3.33*	2	0.69	1.38
Protein fraction (Folch Extr.)	1.29 (N_2_ dried)	1	8.83	8.83

**FIGURE 2:**
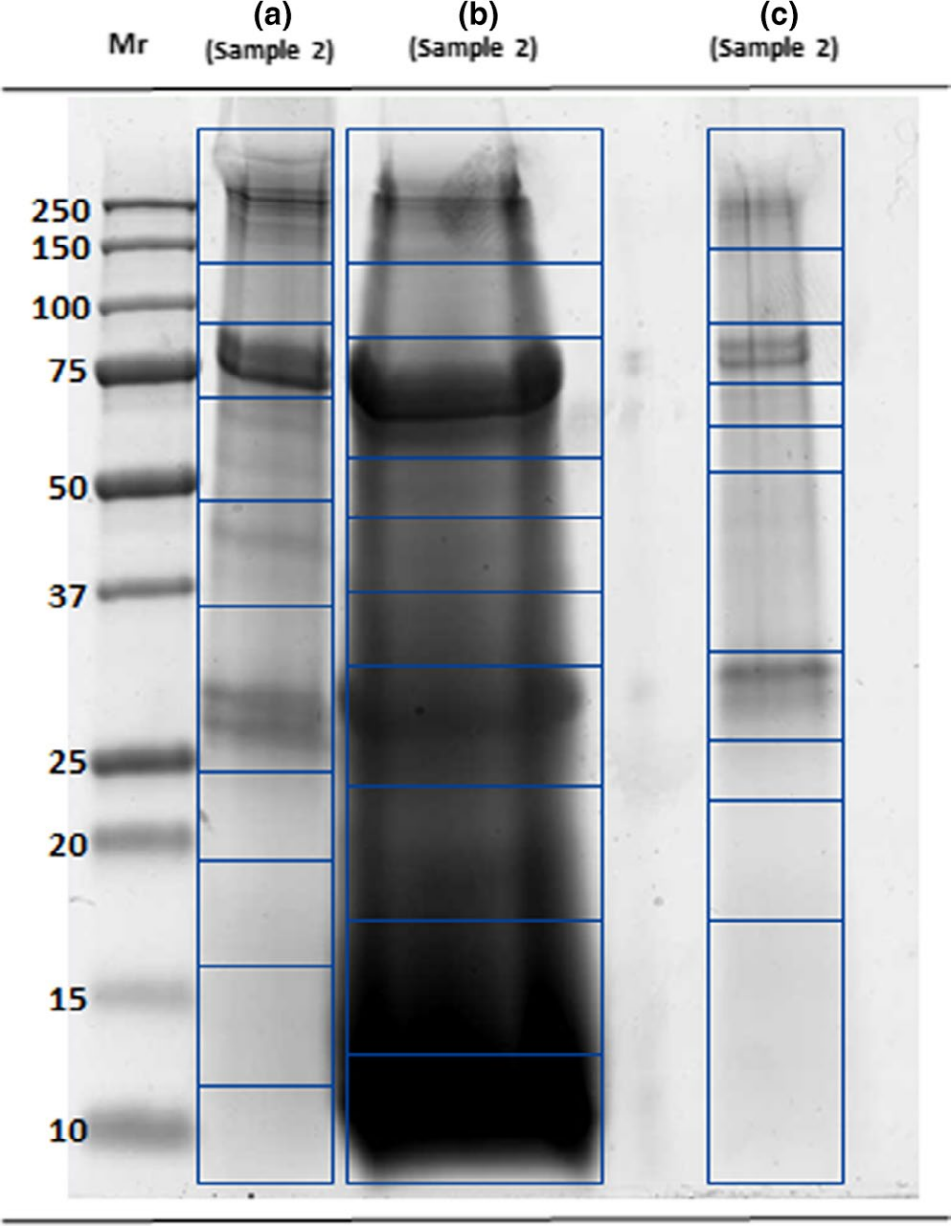
SDS‐PAGE separation of the precipitate (track a), supernatant (track b) obtained by aqueous fractionation versus the protein fraction obtained by Folch extraction (track c). Each lane has been cut into 10 segments, the proteins digested and sent to MS analysis. Mr: molecular mass ladder. Staining with Bio‐Safe Coomassie blue

SDS‐PAGE analyses (Figure 2), in reducing conditions, highlighted the necessity to use all extraction procedure in combination. Coomassie‐stained lanes of the three fractions, respectively, precipitate, supernatant by aqueous fraction and Folch fraction, showed several bands in the molecular weight range with different abundance too. The analyses of all bands from the different lanes generated an in‐depth characterization of *Bombix Mori* proteome by identification of 778 proteins.

Moreover, all the lanes relative to the diverse fractions, except for a few rather intense zones, are characterized by a not‐well‐resolved protein profile, mainly due to the silkworm pupae industrial processing. The not physiological conditions (pH, temperature) during the processes could induce artificial cross‐linking between amino acid residues and their side chains. This could result in the presence of smears into the SDS PAGE.

### 3.2 Qualitative and semiquantitative protein analysis by mass spectrometry

Considering the heterogeneity of the protein profiles obtained by applying different extraction methods it should be noted that every single treatment contributes in the knowledge of the proteome. To achieve an in‐depth comprehensive protein profiling of *Bombix Mori* organism, it was necessary to merge all the results obtained. The merging tool, available among the proteome discoverer options, automatically deletes the redundancies from different tabular reports; it was used to generate the final list of proteins identified in the sample with their protein content normalized (PSMs; Table S1). This list of proteins was used as starting point for functional analyses, as above described.

**FIGURE 3:**
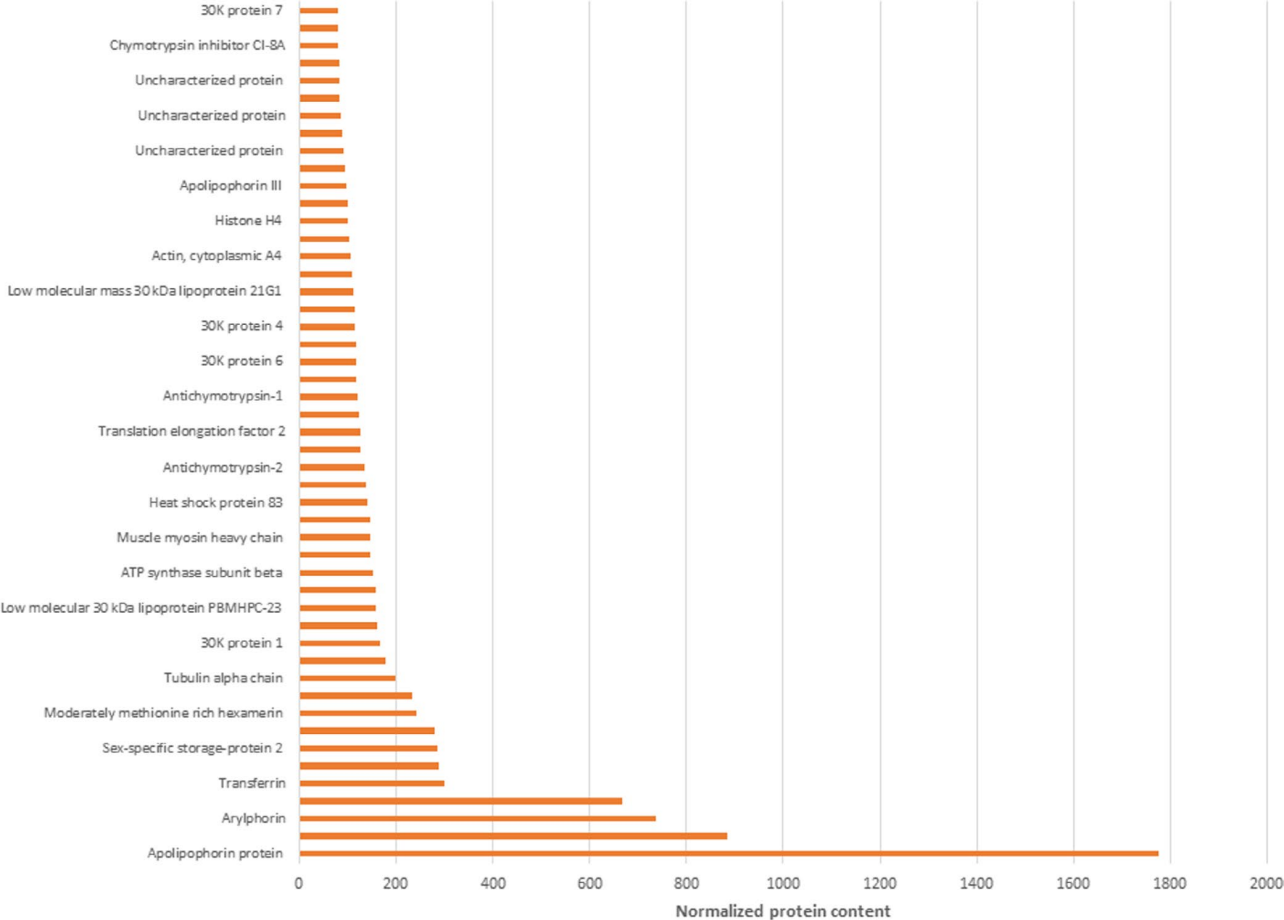
Semiquantitative analysis. The pie chart shows the percentage distribution of reported class of proteins in silkworm pupae processed sample

**FIGURE 4:**
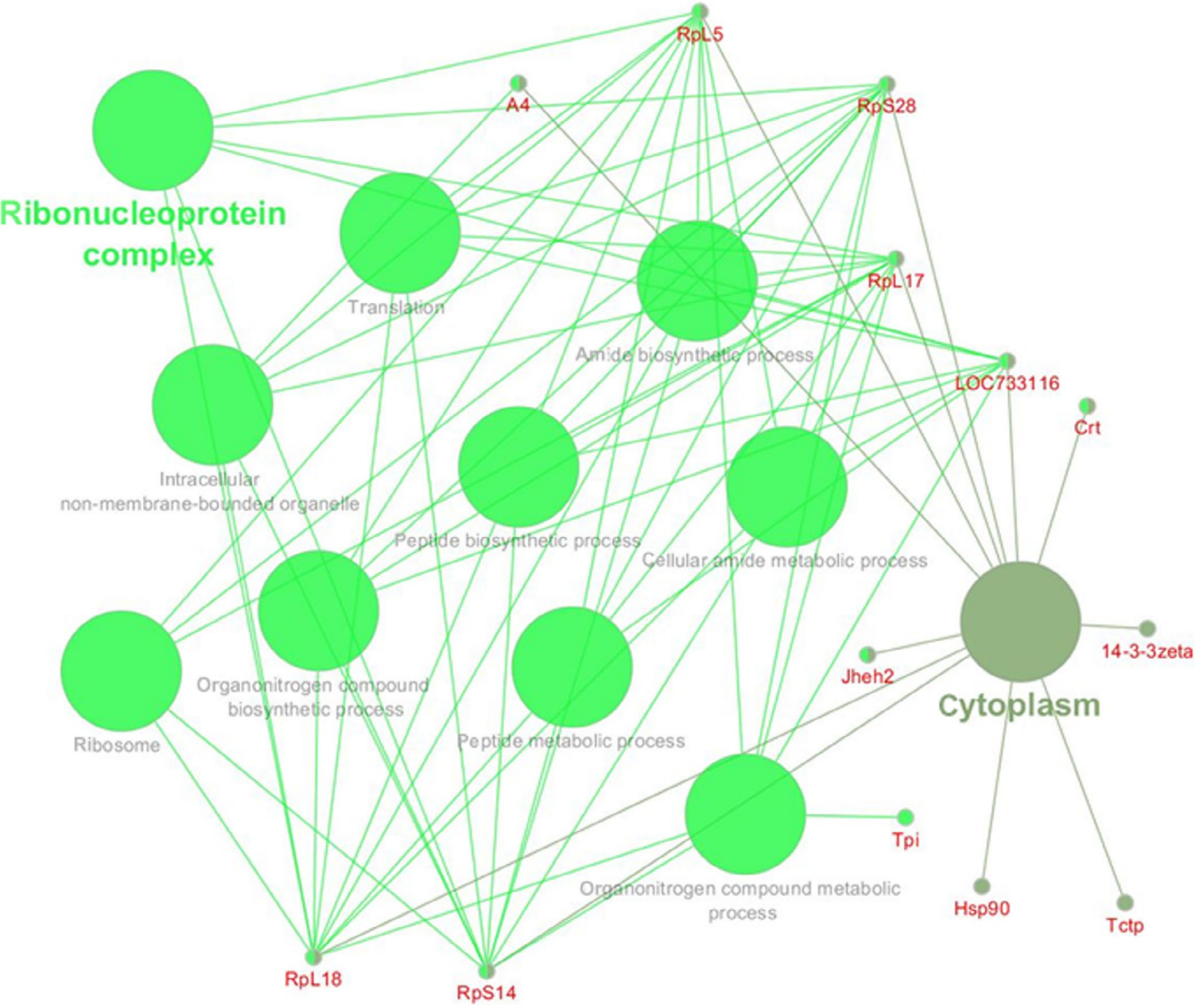
Gene Ontology enrichment by ClueGo in Cytoscape

An estimation of the protein contents in the complex mixtures was obtained by calculating the values of emPAI, normalized by the PSMs values, and add an important quantitative information to proteomic results here shown. Figure 3 depicts the bar plot based on the percentage distribution of reported class of proteins in the sample, obtained by semiquantitative analyses. The most abundant classes of proteins were lipid transfer proteins, as apolipophorin protein and vitellogenin, and sex‐specific storage protein family, as arylphorin and the different isoforms of sex‐specific storage protein (Figure 3). Cytoplasmic proteins, as actin, calreticulin, heat shock proteins, ribosomal proteins, were in‐depth represented, as showed by Gene Ontology analyses too (Figure 4). Known protein allergens resulted also from this characterization: arginine kinase (PMS 146; Liu et al., 2009), 27 kDa glycoprotein (Jeong et al., 2016), thiol peroxiredoxin (PMS 57; Wang et al., 2016), tropomyosin (PMS 139; Jeong et al., 2017), chitinase (PMS 26; Zhao et al., 2015), paramyosin (PMS 41), and Bom m 9 (lipoprotein, PBMHP‐6; PMS 81; Zuo et al., 2015). The protein content normalized of these proteins was lower than the value of the main abundant class of proteins, such as apolipophorin (PMS 1776) and sex‐specific storage protein 1 (PMS 668; Table S1).

### 3.3 Generation of protein–protein interaction network

The protein–protein interaction network here reported (Figure S2) represents the first comprehensive interact‐omics map for the *Bombix Mori* proteome and provides an interesting framework for navigating through the proteome. The topological analysis, thanks to the enrichment tool in STRING, has demonstrated several sparsely connected subnetworks. The analyses, consulting PFAM Protein Domains database, highlighted the class of serpin proteins with highly connected interactions, the lepidopteran low molecular weight (30 kDa) lipoprotein, which represented one of the top protein class in the interact‐omics map, the hemocyanin protein class with a nutrient reservoir activity, the Ras family proteins, or the insect cuticular protein class (chitin‐binding peritrophin‐A domain; Figure S3). While, based on KEGG's database information, the main pathways in which the silkworm proteins are involved were elucidated, as several metabolic, ribosomal, and proteasome activity pathways (Figure S4).

Beside the graphical representation of the clustering analysis (interact‐omics map), the entire proteome has been further inspected, to classify proteins on the basis of their functional annotations (Gene Ontology Annotations), spotlighting the main molecular functions and confirming the interesting biological processes exerted by silkworm pupae (Figure [Link], [Link], [Link]). In “biological process” enrichment analyses (Figure S1—panel A), the largest clusters include proteins involved in biosynthetic/metabolic processes and in the response to stress, data confirmed by observing the KEGG pathway classification (Figure S1—panel D). In “molecular function” enrichment analyses (Figure S1—panel B), the majority of proteins are involved in protein and metal ion binding, while fewer proteins are involved in nucleotides, DNA, and RNA binding. It is also possible to find a great percentage of proteins with catalytic activity. In “intra/extra cellular localization” analyses (Figure S1—panel C), high percentage of cytoplasmic proteins, extracellular proteins, and ribosome proteins were found. Interestingly, the enrichment based on the PFAM domain annotations (Figure S1—panel E), confirms the results of semiquantitative analysis. In particular, lepidopteran low molecular weight lipoproteins, serpins, and hemocyanins were the most enriched protein classes.

### 3.4 Analysis of bioactive peptides after gastrointestinal digestion

To identify bioactive peptides in *Bombix Mori* proteome, the most abundant identified proteins (top 19 proteins in Table S1) were digested “in silico” with stomach and intestinal enzymes. The predicted peptidome resulted in 141 peptide sequences that were investigated to find their potential functional activity. Twenty‐three bioactive peptides were ranked by peptide ranker with score higher than 0.5, using N‐to‐1 neural network probability. Antimicrobial peptides (AMPs) were selected by applying the Discriminate Analysis Classifier score, using CAMP database and considering the geometric means of the *Bioactivity Probability Score* and the *AMP Probability* (Table 2).

**TABLE 2:** Highest score identified peptide sequences with potential bioactivity by Peptide Ranker (http://bioware.ucd.ie/~testing/biowareweb/) and by CAMP (Collection of Anti‐Microbial Peptides) database (http://www.bicnirrh.res.in/antimicrobial/)

Sequence	Results with discriminant analysis classifier			Peptide Ranker—Bioaware			
	Class	Pep. length	AMP probability	Bioactivity probability score	Geometric means	Protein description	Acc. number
DNKDCFL	AMP	7	0.996	0.793914	0.889	Basic juvenile hormone‐suppressible protein 2 [*Bombyx mori*]	H9JH62
NNKMNCM	AMP	7	0.995	0.591135	0.767	30K protein 2 OS = *Bombyx mori*/30K protein 1 [*Bombyx mori*]	E5EVW2/H9J4F6
TREQWF	AMP	6	0.999	0.51067	0.714	30K protein 6 [*Bombyx mori*]	H9J4F5
DNGSGMCK	AMP	8	0.881	0.573337	0.711	actin, muscle‐type A2 [*Bombyx mori*]	H9JWN1
ESCMNCR	AMP	7	0.724	0.689232	0.706	Basic juvenile hormone‐suppressible protein 2 [*Bombyx mori*]	H9JH62
ESCMNCRW	NAMP	8	0.48	0.897656	0.656		
NDNRINF	AMP	7	0.791	0.523558	0.644	Basic juvenile hormone‐suppressible protein 2 [*Bombyx mori*]	H9JH62
RSDCHGF	NAMP	7	0.365	0.814214	0.545		
KDCYTNM	AMP	7	0.531	0.558929	0.545	Silkworm storage protein [*Bombyx mori*]	H9JHM9
DSFDKNL	NAMP	7	0.417	0.529957	0.470		
KNCESCMNC	NAMP	9	0.354	0.533621	0.435		
KNCESCMNCR	NAMP	10	0.209	0.713351	0.386		
QNGKMDF	NAMP	7	0.108	0.61261	0.257		
CMNCRW	NAMP	6	0.054	0.953337	0.227		
DQCIKNF	NAMP	7	0.03	0.665058	0.141		
NCESCMNCRW	NAMP	10	0.021	0.91836	0.139		
NCESCMNC	NAMP	8	0.016	0.643111	0.101		
QRSDCHGF	NAMP	8	0.007	0.778495	0.074		
QTCDMKM	NAMP	7	0.005	0.58367	0.054		
NCESCMNCR	NAMP	9	0.003	0.783407	0.048		
PRGTEGGF	NAMP	8	0.003	0.501702	0.039		
CQSHCRA	NAMP	7	0.002	0.536962	0.033		
SDWRNF	NAMP	6	0.001	0.912881	0.030		

The identified peptide sequences as potential functional peptides have to be validated by further bioactivity assays, using correspondent synthetic peptides. However, these new computational methods are helpful in discovery phase of bioactive peptides. In fact, they are fast and low‐cost alternatives, able to predict and reduce the number of potential targets to be investigated. In addition, bioinformatics‐driven tools provide useful insights not achievable in human or animal model studies.

## 4 DISCUSSION

This is the first study to deeply characterize the high‐value protein content and functional peptides of *B. mori* pupae, an edible insect and waste product of silk industry. This combined analytical approach allowed the identification of 778 proteins and 9,590 sequenced peptides of which 737 with potential functional activities and 18 with antimicrobial activity, as peptides of sequence SPKFCW, DQDPFRP, and PDPSKF. Moreover, semiquantitative analyses showed different value of expressions between protein classes. The main overexpressed class was lipid transport proteins, cytoskeleton, ribosomal proteins, and larval storage protein family (LSP). This class of storage proteins has high‐value content of essential amino acids, and it is used primarily as a source of aromatic amino acids for adult protein synthesis during metamorphosis. In *B. mori*, storage protein 1 (SP1) is constituted by a high number of methionine residues, and storage protein 2 (SP2) of aromatic residues (arylphorin). In the silkworm pupae, these two storage proteins are differentially regulated during larval–pupal transformation, showing lower expression in hemolymph and higher in the fat body (Chen et al., 2015). Storage proteins have several peculiar characteristics, that could be useful for applications. First, sex‐specific storage protein 2 (SSP2), homologous to SP2, is a heat‐resistant protein, able to maintain its biological activity until 80°C. SSP2 have antiapoptotic activity in different cells (Yu, Wang, Zhang, Quan, & Zhang, 2013). In addition, silkworm pupae protein, digested by gastrointestinal endopeptidases, has anti‐inflammatory effect in cells and generates a novel angiotensin‐I‐converting enzyme (ACE) tripeptide inhibitor of sequence ASL, useful in blood pressure control (Qiongying, Junqiang, Hui, Jinjuan, & Zhongzheng, 2015). Finally, *B. mori* releases immune effectors peptides in the hemolymph, after bacterial infections (Romoli et al., 2017).

In this study, peptidomic investigation, supporting by protein profiling, highlighted the presence of several bioactive peptides, originating from edible insects, with antimicrobial activity but also other potential functionality showed by bioinformatic tools.

This analytical approach allowed the selection of potential functional peptides, reducing the number of targets to be investigated by further assays and finally provide useful insights not achievable in human or animal model studies.

The protein profiling showed also the presence of known allergens. Arginine kinase (Bomb 1), an important enzyme involved in growth and development, is the first allergen of silkworm larvae, discovered by using 10 patient plasma (Liu et al., 2009). Another allergen, reported in literature and quantified in this study, is the 27 kDa glycoprotein. This protein, produced in E.Coli, was tested against 15 patient sera and only the 33% reacted (Zhao et al., 2015). The quantified chitinase and paramyosin proteins are also known allergens, verified by immunoproteomics approach ( Jeong et al., 2016). The thiol peroxiredoxin protein of *B. mori* pupae, instead, induces asthma in human, modulating the functions of macrophage cells (Wang et al., 2016). Finally, pupae Bom m 9 (lipoprotein PBMHP‐6) induces asthma in mouse model (Zuo et al., 2015). However, the number of severe outcome due to these allergens and the number of allergic patients is quite limited, due to the low consumption of this insects and the cross reactivity with other allergens that may influence the results. Further studies are needed, and this semiquantitative protein profiling may help in this discovery phase.

This work demonstrated the high value of protein content in *B. mori* pupae, by showing the presence of functional peptides in the gut, by miming the enzymatic digestions. These peptides can have antimicrobial, but also anti‐inflammatory, antioxidative and they can be modulators of immune system. The *B. mori* pupae protein content can be considered functional food ingredients in baked goods, snack products, and meat analogs. The derived bioactive peptides can be produced by sustainable and environmentally friendly processes.

In conclusion, *B. mori* pupae, even if are waste product of silk industry, can have industrial applications as source of high‐value proteins and bioactive peptides in a green and “circular” economy perspective.

## CONFLICT OF INTEREST

The authors declare that they have no conflict of interest that could have influenced the work reported in this paper.

## ETHICAL APPROVAL

The study complies with institutional standards on silkworm research and it was conformed to Directive 2010/63/EU.

## Supporting information

Fig S1A‐B

Fig S1C‐D

Fig S1E

Fig S2

Fig S3

Fig S4

Table S1

Supplementary Material
