# Supplementary figures and images for: Silkworm pupae as source of high‐value edible proteins and of bioactive peptides
Source: Food Sci Nutr. 2020 May 16;8(6):2652–61. doi: 10.1002/fsn3.1546 (PMC7300080; doi:10.1002/fsn3.1546)

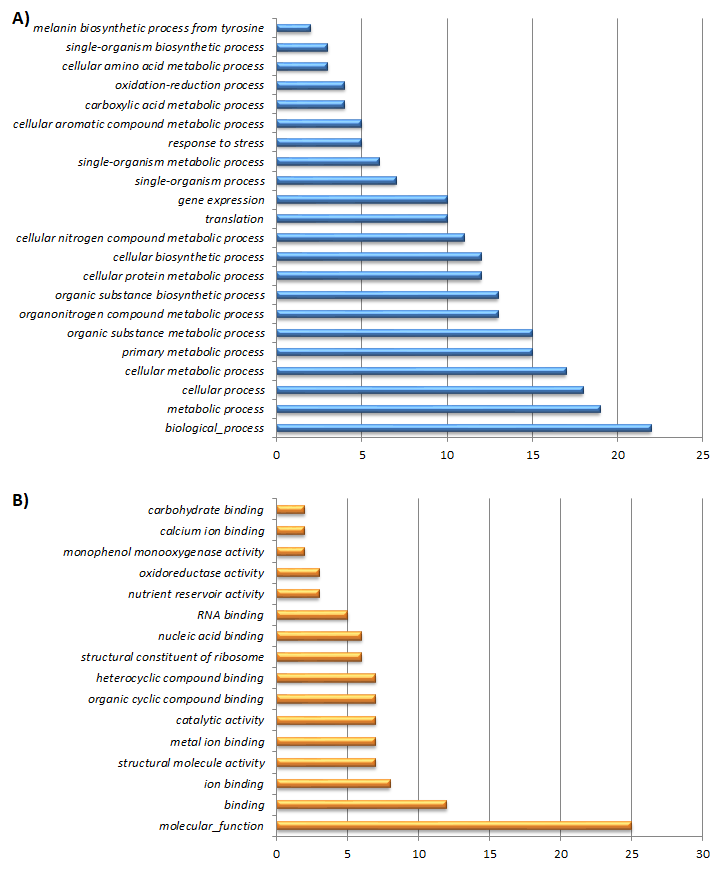

Supplement: Supplementary file 1 — Fig S1A‐B [file FSN3-8-2652-s001.tif]

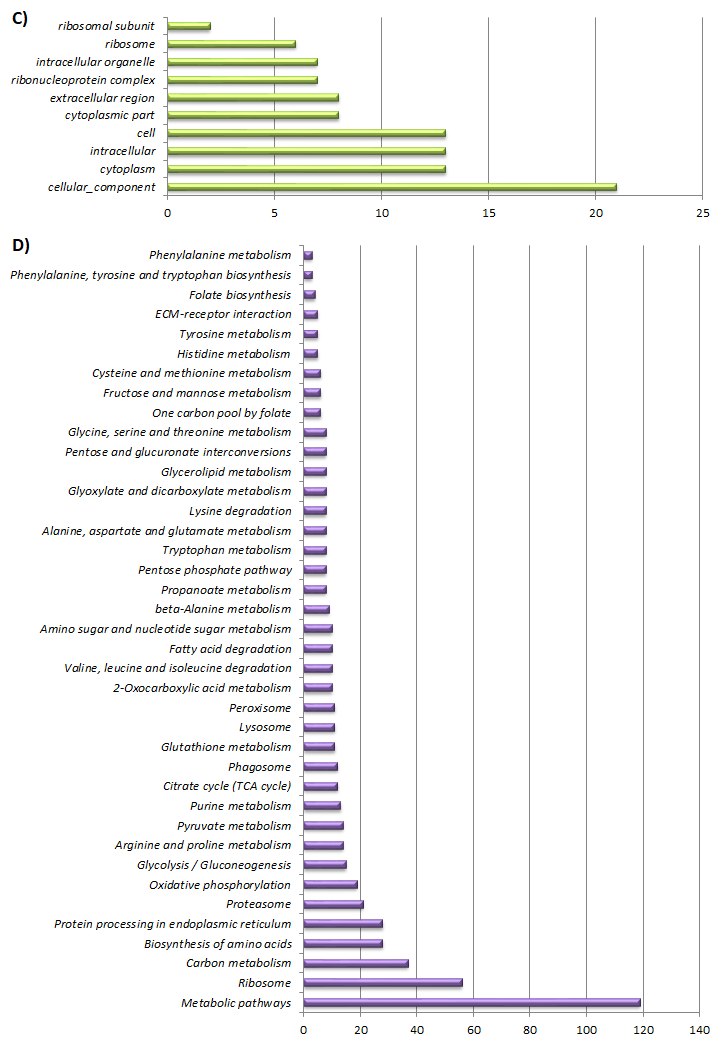

Supplement: Supplementary file 2 — Fig S1C‐D [file FSN3-8-2652-s002.tif]

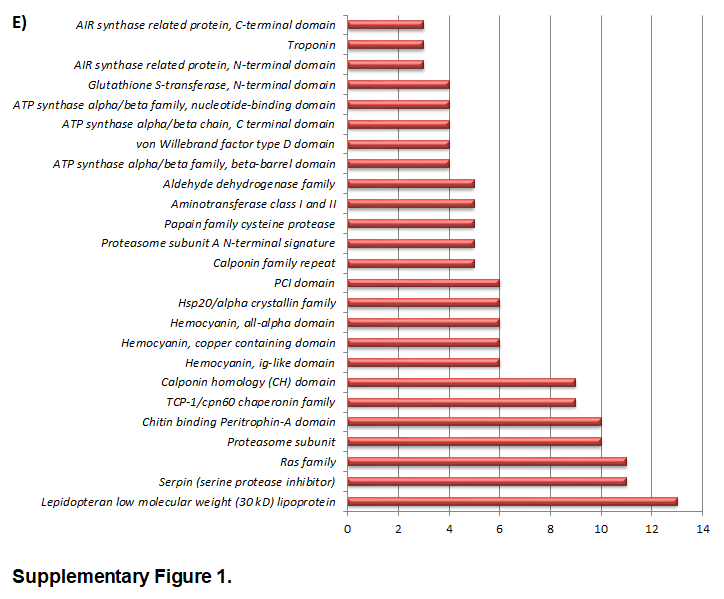

Supplement: Supplementary file 3 — Fig S1E [file FSN3-8-2652-s003.tif]

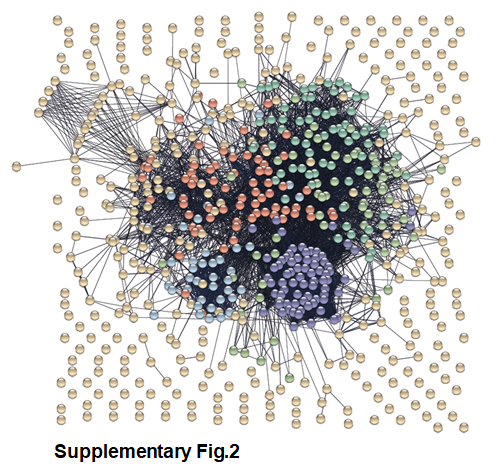

Supplement: Supplementary file 4 — Fig S2 [file FSN3-8-2652-s004.tif]

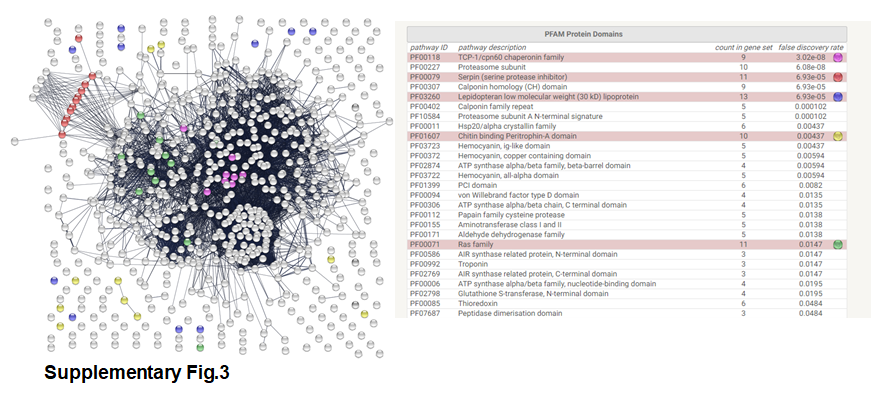

Supplement: Supplementary file 5 — Fig S3 [file FSN3-8-2652-s005.tif]

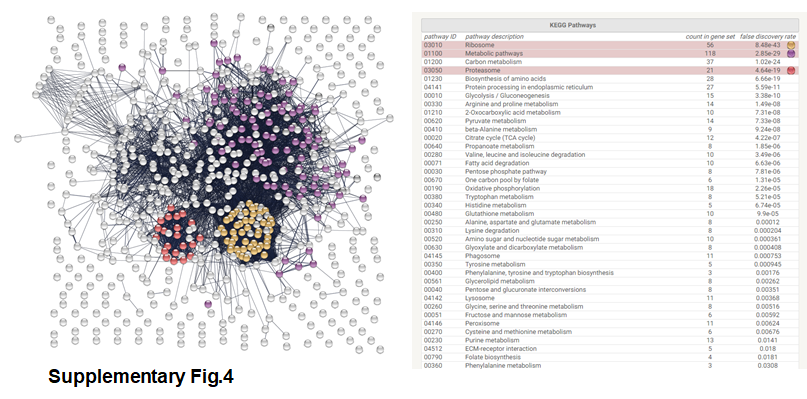

Supplement: Supplementary file 6 — Fig S4 [file FSN3-8-2652-s006.tif]
