# Supplementary material for: Silkworm pupae as source of high‐value edible proteins and of bioactive peptides
Source: Food Sci Nutr. 2020 May 16;8(6):2652–61. doi: 10.1002/fsn3.1546 (PMC7300080; doi:10.1002/fsn3.1546)
Supplement: Supplementary file 8 — Supplementary Material [file FSN3-8-2652-s008.docx]

Figure S1 Gene Ontology analyses based on the biological process (panel A), molecular function (panel B), cellular localization (panel C), KEGG pathway (panel D), and PFAM domain classification (panel E) obtained by using enrichment tool by means of STRING (https://string-db.org/).

Figure S2 Interact-omics map performed by String Analysis. Different colors represent different enriched functions: red nodes are serpin family proteins; green nodes are Ras family proteins; and violet nodes are the lepidopteran low molecular weight (30kDa) lipoprotein.

Figure S3 PFAM enrichment map performed by String Analysis

Figure S4 KEGG enrichment map performed by String Analysis

Table S1 Protein identified in pupae lysate by Proteome Discoverer consulting Uniprot_Bombyx Mori database
